# Uncoupling Protein 3 Promotes the Myogenic Differentiation of Type IIb Myotubes in C2C12 Cells

**DOI:** 10.3390/genes14112049

**Published:** 2023-11-07

**Authors:** Ziwei You, Jieyu Wang, Faliang Li, Wei Hei, Meng Li, Xiaohong Guo, Pengfei Gao, Guoqing Cao, Chunbo Cai, Bugao Li

**Affiliations:** 1 College of Animal Science, Shanxi Agricultural University, 1 Mingxian Nanlu, Jinzhong 030801, China; s20212358@stu.sxau.edu.cn (Z.Y.); z20213450@stu.sxau.edu.cn (J.W.); z20213451@stu.sxau.edu.cn (F.L.); b20211044@stu.sxau.edu.cn (W.H.); mengli@stu.sxau.edu.cn (M.L.); xhguo@sxau.edu.cn (X.G.); gpf800411@sxau.edu.cn (P.G.); sxndcgq@sxau.edu.cn (G.C.); 2 Institute of Animal Sciences, Chinese Academy of Agricultural Sciences, Beijing 100193, China

**Keywords:** C2C12 cells, energy metabolism, myogenic differentiation, types of myotubes, Ucp3

## Abstract

Uncoupling protein 3 (Ucp3) is an important transporter within mitochondria and is mainly expressed in skeletal muscle, brown adipose tissue and the myocardium. However, the effects of Ucp3 on myogenic differentiation are still unclear. This study evaluated the effects of Ucp3 on myogenic differentiation, myofiber type and energy metabolism in C2C12 cells. Gain- and loss-of-function studies revealed that *Ucp*3 could increase the number of myotubes and promote the myogenic differentiation of C2C12 cells. Furthermore, Ucp3 promoted the expression of the type IIb myofiber marker gene myosin heavy chain 4 (*Myh*4) and decreased the expression of the type I myofiber marker gene myosin heavy chain 7 (*Myh*7). In addition, energy metabolism related to the expression of PPARG coactivator 1 alpha (*Pgc*1-*α*), ATP synthase, H^+^ transportation, mitochondrial F1 complex, alpha subunit 1 (*Atp*5*a*1), lactate dehydrogenase A (*Ldha*) and lactate dehydrogenase B (*Ldhb*) increased with *Ucp*3 overexpression. Ucp3 could promote the myogenic differentiation of type IIb myotubes and accelerate energy metabolism in C2C12 cells. This study can provide the theoretical basis for understanding the role of Ucp3 in energy metabolism.

## 1. Introduction

Skeletal muscle plays an important role in maintaining body movement and energy homeostasis. Myofiber is the basic unit of skeletal muscle, which can be divided into four types according to the isomer of myosin heavy chain (Myhc). Myosin heavy chain 7 (*Myh*7), myosin heavy chain 2 (*Myh*2), myosin heavy chain 4 (*Myh*4), and myosin heavy chain 1 (*Myh*1) are the marker genes of type Ι, type ΙΙa, type ΙΙb and type ΙΙx myofibers, respectively [1]. Different myofibers can be transformed into other myofibers [2]. The mitochondrial activity and oxidative metabolism vary in the four types of myofibers. Type Ι myofibers contain a large amount of mitochondria and cytochrome, which promote oxidative metabolism, while the type ΙΙb myofibers are the opposite. The characteristics of type ΙΙa and ΙΙx myofibers are between type Ι and ΙΙb myofibers [3]. The contents of the four types of myofibers are correlated with meat quality. The higher the content of type Ι myofibers, the better the meat quality. Therefore the transformation between different types of myofibers becomes very important in improving meat quality [4].

Uncoupling protein 3 (Ucp3) is an anionic carrier protein located in the inner membrane of mitochondria [5], which is mainly expressed in the skeletal muscles. Ucp3 is expressed more in glycolysis muscle fibers than in oxidized muscle fibers [6]. So far, Ucp3 has mainly been focused on in the research on lipid oxidative metabolism and mitochondrial reactive oxygen species. Ucp3 can promote the transformation of porcine white adipose cells into beige adipose cells, which can promote heat production by increasing the rate of respiratory oxygen consumption in adipose cells [7]. Cai et al. found that the content of subcutaneous fat in *MSTN^−^*^/*−*^ Meishan pigs decreased when the expression of *Ucp*3 was significantly increased [8]. Bezaire et al. found that the skeletal-muscle-specific overexpression of *Ucp*3 mice had stronger fatty acid uptake and oxidation capacity compared with wild-type mice [9]. Similarly, MacLellan et al. indicated that the expression of *Ucp*3 and fatty acid content in rat myoblast cells (L6) increased [10]. Ucp3 can also protect mitochondria from damage caused by oxidative stress [11,12]. However, there are few reports about the effect of Ucp3 on myogenic differentiation, and its function is still unclear.

The present research aimed to explore the regulation of Ucp3 on the myogenic differentiation, myofiber transformation and energy metabolism of C2C12 cells using immunofluorescence staining and a quantitative real-time polymerase link reaction. It provides a theoretical basis for the study of the molecular mechanism of skeletal muscle cell metabolism.

## 2. Materials and Methods

### 2.1. C2C12 Cells Culture and Myogenic Differentiation

The C2C12 cells were purchased from Shanghai Jianing Biotechnology Company. Resuscitated cells were cultured in dishes supplemented with 7 mL of complete medium containing 10% fetal bovine serum (FBS; Gibco, Grand Island, NE, USA, Cat. 10099141). When the cell density reached about 75%, the cells were digested with trypsin (Gibco, Grand Island, NE, USA, Cat. 25200072) and divided into 6-well plates for 24 h of culture. When the cell density reached about 50%, an appropriate amount of lentivirus-packed vectors was added to the serum-free culture medium (Opti-MEM; Gibco, Grand Island, NE, USA, Cat. 31985070) to infect the cells and then cultured for 12 h. Then, the transfected medium was replaced with a complete growth medium (high-glucose DMEM, 10% FBS and 1% penicillin streptomycin) and continued to be cultured for 24 h. When the cells converged to 85%, the transfected C2C12 cells were treated with a culture medium containing 2% horse serum (Gibco, Grand Island, NE, USA, Cat. 16050122) to induce myogenic differentiation. The culture medium was changed every 2 days.

### 2.2. RNA Extraction and cDNA Synthesis

Total RNA was extracted from C2C12 cells using Trizol reagent (Takara, Kusatsu, Japan, Cat. 9108) according to the manufacturer’s instructions. Thereafter, 1 μg of total RNA was reverse transcribed with the PrimeScript Regent Kit with gDNA Eraser (Takara, Kusatsu, Japan, Cat. RR047A) using random hexamer primers according to the manufacturer’s instructions. The total system of the reaction was 20 microliters and was gently mixed and incubated at 50 °C for 5 min and heated at 85 °C for 2 min.

### 2.3. Quantitative Real-Time Polymerase Chain Reaction (qRT-PCR)

Real-time PCR was performed with an Applied Biosystems Quant Studio 3 Real-time PCR System (Thermo Fisher Scientific, Waltham, MA, USA). Quantitative real-time PCR was performed using TB Green Premix Ex Taq II (Takara, Kusatsu, Japan, Cat. RR820A). The expressions of all coding genes were normalized to 18S rRNA. The “2^−ΔΔCt^” formula was used to estimate the mean of the triplicate cycle thresholds (CTs) to obtain the relative expression levels. Gene-specific primers were designed using the online website Primer 3 (http://bioinfo.ut.ee/primer3-0.4.0/ (accessed on 10 August 2023)) and synthesized by Shanghai Shenggong Biotech Co., Ltd. (Shanghai, China). The reaction system was 10 μL and comprised the following: 5 μL SYBR, 4 μL cDNA, and 0.5 μL each for upstream and downstream primers. The reaction procedure was as follows: predenaturated at 95 °C for 30 min, denatured at 94 °C for 10 s, annealed at 60 °C for 20 s, extended at 72 °C for 30 s and carried out for 40 cycles. The primer sequences used for the qRT-PCR analyses are listed in Table 1.

### 2.4. Lentiviral-Mediated Transduction

A pair of short hairpin oligonucleotides (GAAGAGGGCCTTAATGAAAG) targeting the open reading frame (ORF) of *Ucp*3 were designed and synthesized using GenePharma. Both the vector construction and lentivirus package were undertaken using Gene Pharma. The C2C12 cells were cultured in 6-well plates. When the cell density was about 50%, the appropriate amount of lentivirus was directly added to the medium for infection. The culture medium was changed to a fresh medium after 24 h of infection.

### 2.5. Immunofluorescence Staining

After myogenic differentiation for 6 days in C2C12 cells, a large number of myotubes could be observed and immunofluorescence staining was performed. The cells were washed with PBS three times, fixed with 4% paraformaldehyde for 30 min and then cleaned with PBS three times. The cells were permeated using 0.5% Triton X-100 at room temperature for 20 min and sealed with 2% goat serum for 1 h. The cells were then treated with primary antibody and incubated overnight at 4 °C. The information regarding the primary antibody is as follows: anti-Myosin Heavy Chain mouse monoclonal antibody (Myhc, Iowa City, IA, USA, Cat. MF20, 1:100), anti-Myosin Heavy Chain 4 mouse monoclonal antibody (Myh4, Iowa City, IA, USA, Cat. BF-F3, 1:100) and anti-Myosin Heavy Chain 7 mouse monoclonal antibody (Myh7, Iowa City, IA, USA, Cat. BA-D5, 1:100). After incubation with the primary antibody, the cells were washed three times in PBS with 0.025% Tween20 and then incubated with an appropriate fluorescent secondary Goat anti-mouse lgG (H+L) Alexa594 antibody (Chicago, IL, USA, Cat. SA00013-3, 1:100) for 1 h at room temperature. Then, the nuclei were labeled with DAPI. Finally, the cells were washed three times with PBS and observed under a fluorescence microscope (Life Technologies, Brown Deer, WI, USA).

### 2.6. Western Blot

The cells were treated with a lysis buffer supplemented with a protease inhibitor (Solarbio, Beijing, China, Cat. P6730); the total protein was extracted from the cell sample after lysis, which was loaded on 10% SDS-polyacrylamide gel electrophoresis and then transferred onto nitrocellulose filter membranes (Solarbio, Beijing, China, Cat. HATF00010). After the membrane transfer, the membrane was rinsed with PBS and sealed in PBS with 5% skim milk powder for 1 h. Incubating them with primary antibodies, the Ucp3 antibody (1:1000, abclonal, Wuhan, China, Cat. A23285), Myog antibody (1:1000, abclonal, Wuhan, China, Cat. A6664) and Gapdh antibody (1:4000, Proteintech, Wuhan, China, Cat. 60004-1-Ig) were used for a Western blot assay. After washing with TBST, the membranes were incubated with a secondary antibody (1:10,000, LI-COR, Lincoln, NE, USA, Cat. 92632211) at room temperature for 1 h. After the second antibody was incubated, the membranes were exposed using the Odyssey CLX imaging system (LI-COR, Lincoln, NE, USA).

### 2.7. Statistical Analysis

The two groups of samples were compared using an unpaired Student’s t-test, and multiple groups of samples were analyzed using one-way ANOVA. Data were expressed as “means ± SEM”. A value of *p* < 0.05 indicated the difference was significant and *p* < 0.01 represented an extremely significant difference. GraphPad Prism (Version 8, San Diego, CA, USA) was used to conduct the statistical analysis and plotting.

## 3. Results

### 3.1. The Expression Patterns of Ucp3

**Figure 1:**
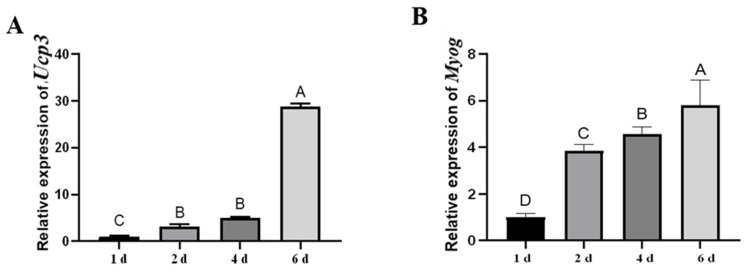
Expression characteristics of *Ucp*3 in myogenic differentiation of C2C12 cells. (**A**,**B**) Timecourse of *Ucp*3 and *Myog* mRNA expression during myogenic differentiation of C2C12 cells. C2C12 cells were differentiated into myoblasts using DMEM, 2% horse serum and 1% double antibody. Bar graphs with the same superscript letters indicate no significant difference (*p* > 0.05), while those with different superscript letters indicate significant differences (*p* < 0.05).

The expression of *Ucp*3 was detected during the myogenic differentiation of C2C12 cells. The expression of *Ucp*3 increased from days 1 to 6, with the highest expression at 6 d (Figure 1A). *Myog* was mainly expressed in the late stage of myogenic differentiation (Figure 1B). The expression patterns of *Ucp*3 were consistent with that of *Myog*, suggesting that Ucp3 may play an important role in the myogenic differentiation of C2C12 cells.

### 3.2. Ucp3 Promoted Myogenic Differentiation of C2C12 Cells

**Figure 2:**
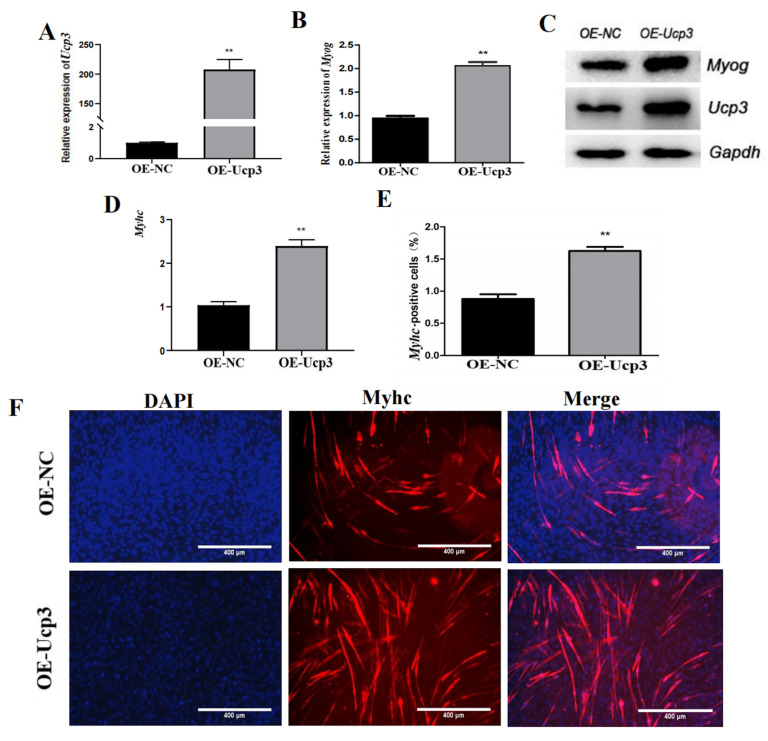
*Ucp*3 overexpression promotes myogenic differentiation in C2C12 cells. (**A**) qRT–PCR was used to test the *Ucp*3 overexpression efficiency in C2C12 cells. (**B**) The expression of Myog gene after affecting the Ucp3 overexpression. (**C**) Expression changes of Ucp3 and myogenic factor Myog at protein level. (**D**) Expression changes in *Myhc* gene after overexpression of *Ucp*3, as determined using qRT–PCR. (**E**,**F**) Myotube formation was detected using immunofluorescence and quantified positive cell statistics. **: *p* < 0.01.

**Figure 3:**
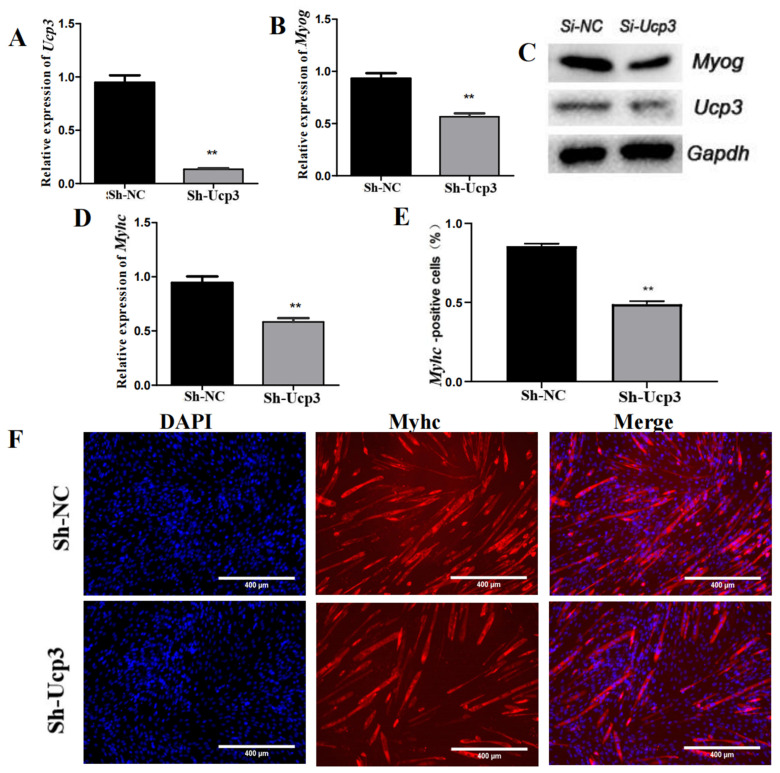
*Ucp*3 silencing slowed myogenesis of C2C12 cells. (**A**) Expression level of *Ucp*3 was determined using qRT–PCR following shRNA knockdown. (**B**) mRNA levels of *Myog* knockdown, as detected using qRT–PCR. (**C**) Western blot was used to test Ucp3 and myogenic factor Myog. (**D**) The level of *Myhc* mRNA after knockdown of *Ucp*3. (**E**,**F**) Myotube formation was detected using immunofluorescence and quantified positive cell statistics. **: *p* < 0.01.

To clarify the function of Ucp3 in myogenic differentiation, the overexpression vector of *Ucp*3 (OE-*Ucp*3) was transfected into C2C12 cells using lentivirus. The expression of *Ucp*3 was significantly increased after transfecting the overexpression vector (Figure 2A). Then, the transfected C2C12 cells were induced to undergo myogenic differentiation. The expressions of *Myog* and *Myhc* were upregulated (Figure 2B,D) and the protein levels of Ucp3 and the myogenic factor Myog were dramatically improved in the Ucp3 overexpression group compared with the control group (Figure 2C). Then, the number of *Myhc* myotubes was increased (Figure 2E,F). In contrast, the mRNA and protein levels of *Ucp*3 and *Myog* were significantly reduced in the C2C12 cells transfected with the *Ucp*3 shRNA vector (Sh-*Ucp*3) (Figure 3A–C). Also, myogenic factor Myhc was significantly downregulated (Figure 3D) and the number of myotubes decreased with the shRNA of Ucp3 (Figure 3E,F).

### 3.3. Effects of Ucp3 on Myofiber Conversion

**Figure 4:**
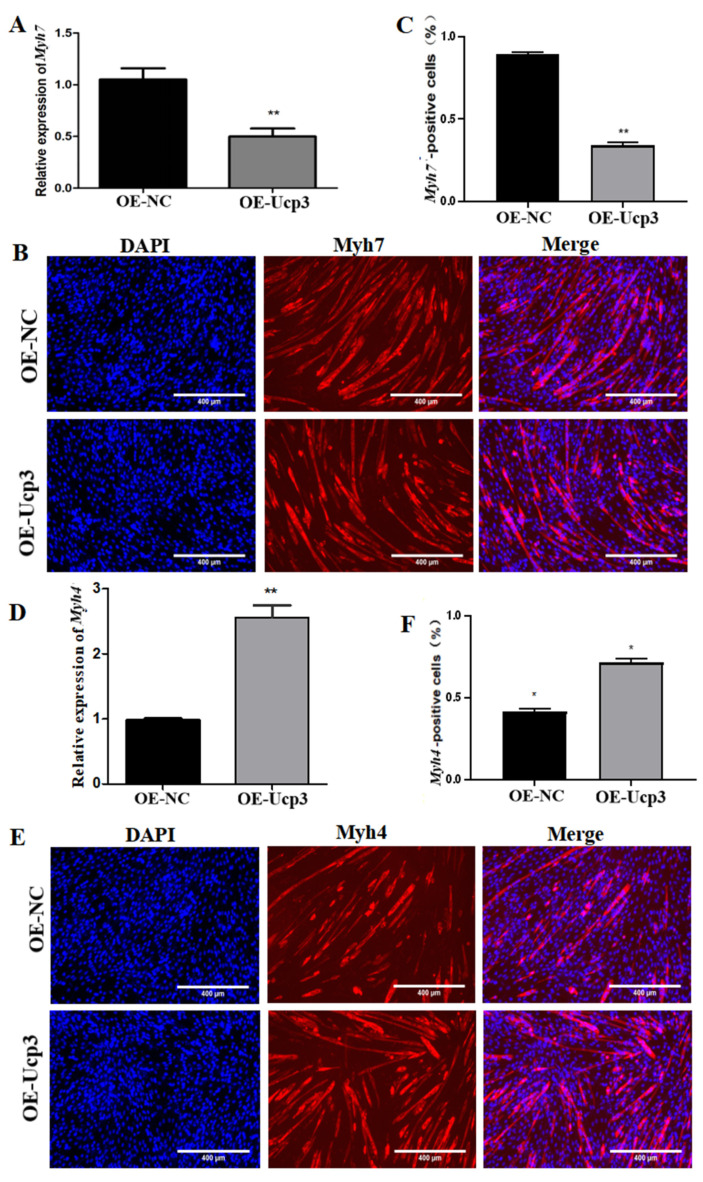
Active *Ucp*3 could motivate the muscle-fiber-type transformation from type I to IIb. (**A**) qRT–PCR was used to test the expression of oxidized muscle fiber marker gene *Myh*7. (**B**,**C**) Immunofluorescence staining was used to identify the positive signal of Myh7. (**D**) The level of colytic muscle fiber marker gene *Myh*4 in C2C12 cells. (**E**,**F**) Immunofluorescence staining was used to identify the positive signal of Myh4. *: *p* < 0.05, **: *p* < 0.01.

**Figure 5:**
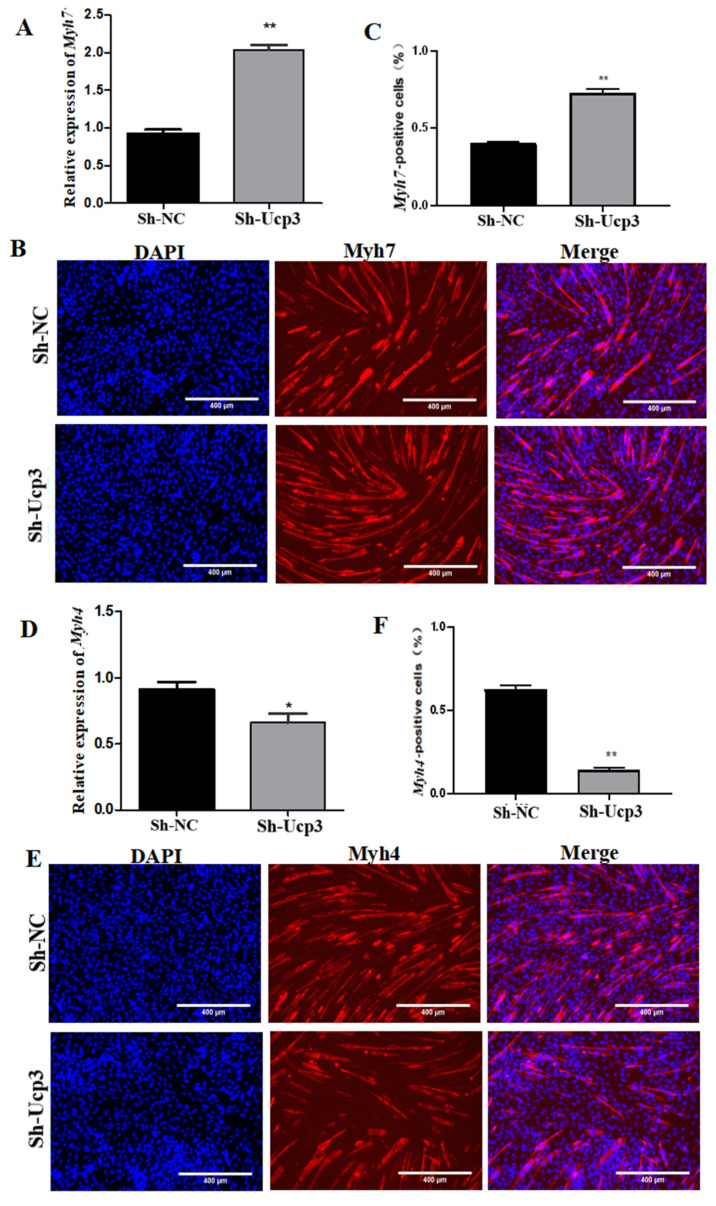
*Ucp*3 knockdown increased the muscle-fiber-type transformation from type IIb to I. (**A**) Expression changes of the oxidized muscle fiber marker gene *Myh*7 were determined using qRT–PCR following inhibitor of *Ucp*3. (**B**,**C**) Myotube formation was detected using immunofluorescence under the same conditions and quantified using positive cell statistics. (**D**) Expression changes of the colytic muscle fiber marker gene *Myh*4 using qRT–PCR, as specified in the legend. (**E**,**F**) Positive signal identification assay carried out in the same way as in panel. *: *p* < 0.05, **: *p* < 0.01.

To further determine the regulatory effect of Ucp3 on myofiber types during myogenic differentiation, the overexpression vector of *Ucp*3 was transfected into C2C12 cells using lentivirus. The expression of *Myh*7 decreased (Figure 4A) and the number of *Myh*7-positive cells reduced (Figure 4B,C). Meanwhile, the expression of *Myh*4 was upregulated (Figure 4D) and the number of *Myh*4-positive cells increased (Figure 4E,F). In contrast, the expression of *Myh*7 was upregulated (Figure 5A) and the number of *Myh*7-positive cells increased (Figure 5B,C) in C2C12 cells transfected with the *Ucp*3 shRNA vector. The result of *Myh*4 was the opposite of *Myh*7 (Figure 5D–F).

### 3.4. Regulation Effects of Ucp3 on Energy Metabolism in C2C12 Cells

**Figure 6:**
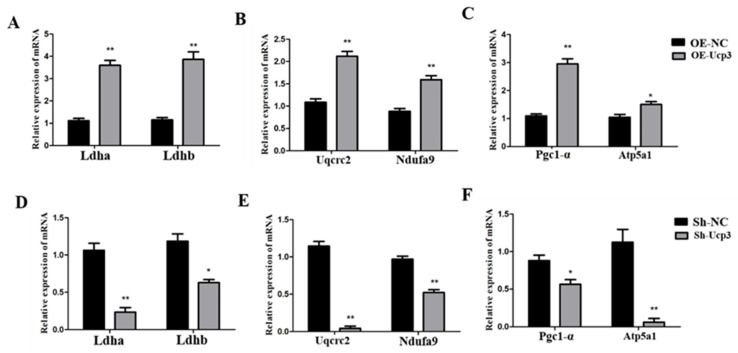
*Ucp*3 had a positive regulatory effect on energy metabolism in C2C12 cells. (**A**–**C**) Relative levels of genes associated with energy metabolism due to overexpression of *Ucp*3 in C2C12 cells. (**D**–**F**) Quantitative real-time PCR analysis of gene expression levels associated with energy metabolism by silencing *Ucp*3 gene. *: *p* < 0.05, **: *p* < 0.01.

Subsequently, the effects of Ucp3 on energy metabolism were detected. With the *Ucp*3 overexpression in C2C12 cells, the expression of the glycolysis-related genes (*Ldha* and *Ldhb*), oxidative-phosphorylation-related genes (*Uqcrc*2 and *Ndufa*9) and mitochondrial-activity-related genes (*Pgc*1-*α* and *Atp*5*a*1) increased significantly (Figure 6A–C). In contrast, all the above gene expressions decreased due to *Ucp*3 knockdown (Figure 6D–F).

## 4. Discussion

Ucp1, Ucp2 and Ucp3 are the members of the Ucp family. Ucp1 is mainly enriched in brown adipose tissue, which is the key protein for nonshivering thermogenesis in mammals. Ucp1 plays an important role in regulating energy metabolism and mitochondrial homeostasis by uncoupling oxidation and phosphorylation, which can consume the energy of the proton gradient in the electron chain to generate heat [13]. Ucp2 is expressed in a variety of tissues, such as white adipose tissue, skeletal muscle, heart, spleen, lung and thymus, which participate in various metabolic processes, including vascular diseases [14], fatty acid metabolism [15], inflammation and oxidative stress [16,17]. Ucp3 is mainly found in skeletal muscle, brown adipose tissue and the myocardium [18,19,20]. Ucp3 plays an important role in regulating energy metabolism, thermogenesis and lipid metabolism in animals. The growth hormone (GH) upregulates the expression of *Ucp*3 gene, thus increasing the heat production of the body [21]. Kerstin et al. reported that a hamster lacking *Ucp*3 in brown adipose tissue displays reduced cold tolerance and has impaired nonshivering thermogenesis [22]. Furthermore, *Ucp*3 expression was significantly increased in the subcutaneous adipose tissue of pigs under cold conditions, and the expressions of beige adipose marker genes (*Cd*137 and *Tmem*26) and thermogenesis marker genes (*Pgc*1-*α*, *Prdm*16 and *Cidea*) were significantly increased, along with the enhancement of mitochondrial activity [7]. Ucp3 is involved in the production of mitochondrial reactive oxygen species (ROS) to prevent mitochondrial oxidative damage [23].

Ucp3 plays an important role in regulating the growth development of skeletal muscle. The expression of *Ucp*3 in the skeletal muscle of hypothyroidism rats is significantly lower than that of rats with normal thyroid function [24]. Ucp3 is strongly associated with Ca^2+^ concentration in skeletal muscle. Recent studies highlighted that an intracellular Ca^2+^ increase can promote the phosphorylation of Camk2, which enhances the *Ucp*3 expression and fatty acid oxidation [25]. In addition, maternal exercise regulates the content of Ca^2+^, activating the apelin-AMPK signaling pathway, which promotes the expression of *Ucp*3 in fetal skeletal muscle [26]. Previous studies showed that miR-181a decreases ATP production in vivo by targeting IGFBP to downregulate the expression of *Ucp*3 in rat skeletal muscle [27]. At present, there are few reports on the regulation of Ucp3 during the proliferation and differentiation of myoblasts. Gynostemma pentaphyllum extract (GPE) and Gypenoside L (GL) promote the myogenic differentiation of C2C12 cells via activation of the PGC-1α pathway and stimulate the expression of *Ucp*2 and *Ucp*3, thus enhancing the anti-oxidative stress ability of the body [28]. Branched-chain amino acid (BCAA) is a key regulator of protein synthesis in skeletal muscle, which promotes the myogenic differentiation of C2C12 cells by activating mTORC1 signaling pathways and significantly increasing the expression of *Ucp*3 [29]. Furthermore, Yukiko et al. demonstrated that the expression of *Ucp*3 shows an upward trend during the myoblast differentiation of C2C12 cells, with the highest expression on the eighth day, indicating that Ucp3 may play an important role in the later stage of myoblast differentiation [30]. Our data show that Ucp3 promoted the myoblastic differentiation of C2C12 cells.

Ucp3 plays a key role in controlling energy metabolism in skeletal muscle. Past studies showed that the expression of *Ucp*3 is elevated in acute exercise and decreased in endurance exercise in skeletal muscle [31]. Compared with normal mice, *Ucp*3^−/−^ mice show a deficiency in ATP production, oxygen consumption and heat production [32]. Curcumin significantly attenuates myocardial apoptosis in rat cardiomyocytes, and the expression of *Ucp*3 decreases, indicating that Ucp3 may be involved in curcumin-mediated mitochondrial protection [33]. Additionally, the expression of *Ucp*3 and *Glut*4 increases in lanthionine synthase C-like (*LANCL*1)-overexpressing L6 cells, and oxygen consumption is obviously increased [34]. Insulin-like growth factor-1 (IGF-1) upregulates *Ucp*3 expression via the phosphorylation of Foxo4, and *Ucp*3 expression is significantly reduced after the addition of PI3K inhibitors, demonstrating that IGF-1 plays a role in energy homeostasis by regulating *Ucp*3 expression in C2C12 myoblasts through the PI3-Akt/foxo4 pathway [30]. Moreover, this study showed that Ucp3 contributes to the expression of glycolysis key genes (*Ldha*, *Ldhb*), oxidative phosphorylation metabolic genes (*Uqcrc*2, *Ndufa*9) and mitochondrial activity marker genes (*Pgc*1-*α*, *Atp*5*a*1), thereby regulating energy metabolism in C2C12 cells.

The conversion of skeletal muscle fiber types is closely related to energy metabolism. Pgc-1α transgenic pigs increase skeletal muscle mitochondrial biogenesis and ATP synthesis, along with the upregulation of oxidative fiber markers and downregulation of glycolytic fiber markers [35]. Similarly, endurance training in mice enhances skeletal muscle mitochondrial activity through activation of the AMPK signaling pathway, leading to the conversion of type ΙΙb muscle fibers to type Ι muscle fibers [36]. *Ucp*3 is mainly expressed in type ΙΙb muscle fibers and less so in type Ι muscle fibers [37]. Anguer et al. found that mice overexpressing *Ucp*3 significantly increase oxygen consumption and oxidize fiber markers after endurance training [38]. In addition, the number of type ΙΙb myofibers in the skeletal muscle of ovariectomized mice increases significantly and the expression of *Ucp*3 also increases [39]. Moreover, by interfering with the *Ucp*3 gene in porcine skeletal muscle myoblasts, the activity of Ldh decreases as the expression of type ΙΙb myofiber marker gene *Myh*4 decreases and the expression of type Ι myofiber marker gene *Myh*7 increases [40]. The same result was obtained in this study. The expression of the type IIb myofiber marker gene *Myh*4 was significantly reduced and the number of myotubes was also reduced in C2C12 cells after interfering with *Ucp*3. Meanwhile, the expression of the type I myofiber marker gene *Myh*7 was opposite to that of *Myh*4.

There were some limitations to this study. This study found that Ucp3 could promote myogenic differentiation and accelerate the conversion of myofibers from type Ι to type ΙΙb in C2C12 cells. But, the molecular mechanism of Ucp3 in myogenic differentiation, myotube fusion and energy metabolism is missing. Further research is needed. All experiments in this study were only completed on C2C12 cells. More experiments should be done in other cells, such as mouse, pig, and human primary myoblasts and cell lines. The experimental method was relatively limited in this study. The mRNA expression data and immunofluorescence data presented were insufficient to support the conclusion. The expression of mRNA could only reflect the gene transcription, and could not indicate the amount of protein content. In many cases, the data of mRNA expression and protein content was not consistent because of the post-transcriptional regulation. The immunofluorescence data of protein may not accurately reflect the changes in protein content due to the non-specific immune response. A Western blot experiment can eliminate the band of non-specific immune response through the size of the protein. A Western blot experiment should be done to accurately reflect the changes in protein content. The Western blot experiment successfully obtained the bends of UCP3 and myogenin but failed on Myh4 and Myh7. Therefore, more experiments should be done in further studies.

In my opinion, Ucp3 can promote myotube fusion via the conversion of the mitochondrial inner membrane potential difference into heat in the late stage of the myogenic differentiation of C2C12 cells. Overall, the myogenic differentiation of C2C12 cells requires ATP and heat. PGC1a can enhance cellular respiration and lead to an increase in the mitochondrial inner membrane potential by activating the AMPK and mTOR signaling pathways during the late stage of myogenic differentiation [26,28,29,41]. The high potential of the mitochondrial inner membrane causes ATP synthase to produce ATP while generating heat using Ucp3. Therefore, cellular respiration, mitochondrial activity and Ucp3 content were significantly enhanced during the myogenic differentiation of C2C12 cells. The substrate for cellular respiration is NADH, which is produced via glycolysis and the tricarboxylic acid cycle (TCA cycle) while generating some ATP. Therefore, glycolysis is also significantly enhanced during the myogenic differentiation of C2C12 cells.

## 5. Conclusions

Ucp3 could promote myogenic differentiation in C2C12 cells and accelerate the conversion of myofibers from type Ι to type ΙΙb. The genes for glycolysis, oxidative phosphorylation and mitochondrial activity increased with *Ucp*3 overexpression.

## Figures and Tables

**Table 1:** Primer sequences.

Gene	Primer Sequences (5′–3′)
*Ucp*3	F: GCCGGCACTGCGGCCTGTTTT R: TGTGCGCACCATAGTCAGGAT
*Myog*	F: TCCCAACCCAGGAGATCATT R: AGTTGGGCATGGTTTCGTCT
*Myhc*	F: ACTTGTGGTGTCGGTCACTC R: CTGAAAATCAGCCGCACGTC
*Myh*4	F: CTCACCTACCAGACCGAGGA R: CTCCTGTCACCTCTCAACAGA
*Myh*7	F: GATTCCTCTAGGACAGCAGCG R: TTCCTTTCTCTGAGCCACCTTG
*Pgc*1-*α*	F: TGTGTGCTGTGTGTCAGAGT R: ACCAGAGCAGCACACTCTAT
*Atp*5*a*1	F: TTGTTGGTGCAAGAAATCTCCA R: TACCATCACCAATGCTTAACACA
*Ldha*	F: AACTTGGCGCTCTACTTGCT R: GGACTTTGAATCTTTTGAGACCTTG
*Ldhb*	F: AAAGGCTACACCAACTGGGC R: GCCGTACATTCCCTTCACCA
*Uqcrc*2	F: CCGGGTCCTTCTCGAGATTTT R: TGCTTCAATCCCACGGGTTA
*Ndufa*9	F: TTCCAATGTCACGTCCTGCC R: CTTGTGACCCCATTCGTCCA
18*S rRNA*	F: ATAAACGATGCCGACTGGCGAT R: CAATCTGTCAATCCTGTCCGTGT

F: forward; R: reverse.

## Data Availability

All data generated or analyzed during this study are included.
